# Per- and polyfluoroalkyl substances (PFAS) in mixtures show additive effects on transcriptomic points of departure in human liver spheroids

**DOI:** 10.1093/toxsci/kfad044

**Published:** 2023-05-17

**Authors:** Gregory C Addicks, Andrea Rowan-Carroll, Anthony J F Reardon, Karen Leingartner, Andrew Williams, Matthew J Meier, Ivy Moffat, Richard Carrier, Luigi Lorusso, Barbara A Wetmore, Carole L Yauk, Ella Atlas

**Affiliations:** Environmental Health Science and Research Bureau, Healthy Environments and Consumer Safety Branch (HECSB), Health Canada, Ottawa, Ontario K1A 0K9, Canada; Environmental Health Science and Research Bureau, Healthy Environments and Consumer Safety Branch (HECSB), Health Canada, Ottawa, Ontario K1A 0K9, Canada; Environmental Health Science and Research Bureau, Healthy Environments and Consumer Safety Branch (HECSB), Health Canada, Ottawa, Ontario K1A 0K9, Canada; Environmental Health Science and Research Bureau, Healthy Environments and Consumer Safety Branch (HECSB), Health Canada, Ottawa, Ontario K1A 0K9, Canada; Environmental Health Science and Research Bureau, Healthy Environments and Consumer Safety Branch (HECSB), Health Canada, Ottawa, Ontario K1A 0K9, Canada; Environmental Health Science and Research Bureau, Healthy Environments and Consumer Safety Branch (HECSB), Health Canada, Ottawa, Ontario K1A 0K9, Canada; Water and Air Quality Bureau, HECSB, Health Canada, Health Canada, Ottawa, Ontario K1A 0K9, Canada; Water and Air Quality Bureau, HECSB, Health Canada, Health Canada, Ottawa, Ontario K1A 0K9, Canada; Chemicals and Environmental Health Management Bureau, Healthy Environments and Consumer Safety Branch (HECSB), Health Canada, Ottawa, Ontario K1A 0K9, Canada; Center for Computational Toxicology and Exposure, Office of Research and Development, U.S. Environmental Protection Agency, Research Triangle Park, North Carolina 27711, USA; Department of Biology, University of Ottawa, Ottawa, Ontario K1N 6N5, Canada; Environmental Health Science and Research Bureau, Healthy Environments and Consumer Safety Branch (HECSB), Health Canada, Ottawa, Ontario K1A 0K9, Canada; Department of Biochemistry, University of Ottawa, Ottawa, Ontario K1H 8M5, Canada

**Keywords:** TempO-Seq, PFAS, liver spheroids, benchmark concentration, new approach methodology, mixture toxicity

## Abstract

Per- and polyfluoroalkyl substances (PFAS) are a wide range of chemicals that are used in a variety of consumer and industrial products leading to direct human exposure. Many PFAS are chemically nonreactive and persistent in the environment, resulting in additional exposure from water, soil, and dietary intake. While some PFAS have documented negative health effects, data on simultaneous exposures to multiple PFAS (PFAS mixtures) are inadequate for making informed decisions for risk assessment. The current study leverages data from previous work in our group using Templated Oligo-Sequencing (TempO-Seq) for high-throughput transcriptomic analysis of PFAS-exposed primary human liver cell spheroids; herein, we determine the transcriptomic potency of PFAS in mixtures. Gene expression data from single PFAS and mixture exposures of liver cell spheroids were subject to benchmark concentration (BMC) analysis. We used the 25th lowest gene BMC as the point of departure to compare the potencies of single PFAS to PFAS mixtures of varying complexity and composition. Specifically, the empirical potency of 8 PFAS mixtures were compared to predicted mixture potencies calculated using the principal of concentration addition (ie, dose addition) in which mixture component potencies are summed by proportion to predict mixture potency. In this study, for most mixtures, empirical mixture potencies were comparable to potencies calculated through concentration addition. This work supports that the effects of PFAS mixtures on gene expression largely follow the concentration addition predicted response and suggests that effects of these individual PFAS in mixtures are not strongly synergistic or antagonistic.

Per- and polyfluoroalkyl substances (PFAS) are a large category of synthetic fluorinated chemicals with unique properties that make them attractive for numerous applications. Some types of PFAS have extraordinary thermal and chemical stability and unique hydrophobic and lipophobic properties which, while useful for their applications, also convey long-term environmental stability and facilitate environmental distribution (Gaines *et al.*, 2023). PFAS have been detected worldwide in both humans (Hansen *et al.*, 2001; Kubwabo *et al.*, 2004) and wildlife (Giesy and Kannan, 2001; Wania, 2007). Human exposure routes may include consumer products (Gaines, 2023), diet (Fromme *et al.*, 2007; Vestergren *et al.*, 2012), drinking water (Hu *et al.*, 2016; Health Canada, 2018a,b) and household dust (De Silva *et al.*, 2012; Kubwabo *et al.*, 2005; Shoeib *et al.*, 2011). The effects of several PFAS have been extensively studied in animal models and demonstrate major impacts on the liver, including steatosis and carcinogenesis (Das *et al.*, 2017). Documented human health effects of PFOS and PFOA exposures are extensive (Fenton *et al.*, 2021) and include impaired liver function (Bassler *et al.*, 2019; Costello *et al.*, 2022; Jain and Ducatman, 2019; Salihovic *et al.*, 2018; Sen *et al.*, 2022) and other metabolic effects such as altered lipid metabolism and blood lipid levels (Fisher *et al.*, 2013; Geiger *et al.*, 2014; Jain and Ducatman, 2018; Steenland *et al.*, 2009).

Regulatory agencies are developing guidelines for PFAS and thus need to identify exposure levels that may pose health hazards. Extensive data exist for legacy PFAS (eg, perfluorooctanoic acid [PFOA] and perfluorooctanesulfonic acid [PFOS]) and guidelines based on their administered equivalent doses have been proposed in Canada and elsewhere (Health Canada, 2018a,b). Although PFOS and PFOA are now regulated (Health Canada, 2006, 2017a,b) and no longer manufactured in North America, replacement PFAS have been introduced and thousands of other PFAS are currently produced worldwide (OECD/UNEP Global PFC Group, 2018). Toxicity data are lacking for thousands of PFAS; thus, new approach methodologies (NAMs) (Kavlock *et al.*, 2018) are being employed to increase throughput and decrease cost in toxicity testing for these chemicals. The goal of the NAM application presented herein is to generate gene expression data to determine the cellular exposure levels that result in gene expression perturbations that may be indicative of an adverse biological response. These data may be used for *in vitro* to *in vivo* extrapolation to identify potential risks for various routes of external PFAS exposure, and for modeling potencies of additional PFAS with similar characteristics to those tested.

Two previous papers from our group analyzed the transcriptomic effects of 23 PFAS in human primary liver cell spheroids (Reardon *et al.*, 2021; Rowan-Carroll *et al.*, 2021). A multidonor human primary liver cell spheroid model was chosen for these analyses as PFAS exposure has been consistently associated with altered liver functions (Bassler *et al.*, 2019; Costello *et al.*, 2022; Jain and Ducatman, 2019; Salihovic *et al.*, 2018; Sen *et al.*, 2022). Additionally, PFAS have been implicated in the activation of metabolism regulating nuclear receptors, including peroxisome proliferator-activated receptor alpha (PPARα), that are expressed in liver cells (Behr *et al.*, 2020; Bjork *et al.*, 2011). Based on potential adverse liver effects of PFAS, the liver was identified as a critical target organ in Health Canada’s regulatory guidelines on PFAS (Health Canada, 2018a,b). Although the potential negative health effects of PFAS are extensive, the toxicological mode(s) of action of PFAS may be complex with individual PFAS potentially having multifarious mechanisms (Fenton *et al.*, 2021; Fragki *et al.*, 2021). In our previous work (Reardon *et al.*, 2021; Rowan-Carroll *et al.*, 2021), we compared transcriptomic changes across different PFAS as a measure of toxicity that can be interpreted independently of mechanistic, or mode of action, analysis; instead, transcriptional changes were used as a generalized method for toxicological assessment representing the concentration at which a robust baseline change in molecular regulation occurs (Johnson *et al.*, 2022).

The present study leverages our existing transcriptomic database of concentration-response studies in human liver cell spheroids exposed to individual PFAS to investigate the effects of PFAS in mixtures. For this analysis, we are assuming that PFAS largely act through similar modes of action. In such cases, mixture potency can be predicted through the concentration addition paradigm (also called dose addition) with individual chemical concentrations and potencies combining to result in a sum total effect equal to the effects of the individual chemical components (Berenbaum, 1985). In rare but important exceptions, chemical mixtures may have synergistic or antagonistic effects with a portion of mixture components causing changes that result in other mixture components having strongly amplified or blunted effects (Kortenkamp and Faust, 2018; Martin *et al.*, 2021). Herein, we determine if PFAS exhibit additive effects, as predicted by concentration addition on transcriptomic points of departure (tPODs), when present in mixtures following short-term (24 hour) and long-term (10 day) exposures. Considering the widespread distribution and long environmental life of PFAS, exposures to multiple PFAS is a global concern and understanding their action as mixtures is a necessary step in forming evidence-based regulatory guidelines for PFAS chemicals.

## Materials and methods

*Note*: This study is part of an ongoing multipart project that is focused on transcriptomic effects of PFAS exposures. Two companion articles, one foundational study and one study with an expanded set of PFAS, that focus on individual PFAS exposures have been previously published (Reardon *et al.*, 2021; Rowan-Carroll *et al.*, 2021). The subset of PFAS used for this mixture-focused analysis was selected in order to include the prototypical PFAS: PFOA, PFOS, and several related carboxylate (PFCAs)- and sulfonate (PFSAs)-type PFAS of varying fluorinated carbon chain length. These included 8 PFCAs: perfluorobutanoate (PFBA)—C4, perfluoropentanoate (PFPeA)—C5, perfluorohexanoate (PFHxA)—C6, perfluoroheptanoate (PFHpA)—C7, perfluorooctanoate (PFOA)—C8, perfluorononanoate (PFNA)—C9, perfluorodecanoate (PFDA)—C10, and perfluoroundecanoate (PFUnA)—C11; and 3 PFSAs: perfluorobutane sulfonate (PFBS)—C4, perfluorohexane sulfonate (PFHxS)—C6, and perfluorooctane sulfonate (PFOS)—C8. Additionally, other common PFAS, 6:2 fluorotelomer sulfonate (6:2 FtS) and 8:2 fluorotelomer sulfonate (8:2 FtS) and perfluorooctane sulfonamide (PFOSA) were included. The structures and CAS numbers of all PFAS used in this study are available in Supplementary Table 1.

### Cell culture

3D InSight Human Liver Microtissues were purchased from InSphero (Brunswick, Maine) in a 96-well format, with a single spheroid per well. These spheroids are a coculture model from 10 different human liver donors including males and females and are a metabolically active system of hepatocytes and Kupffer cells (Proctor *et al.*, 2017; Rowan-Carroll *et al.*, 2021). Upon arrival, culture media were replaced with InSphero Human Liver Maintenance Medium–Tox (InSphero ), and spheroids were acclimated at 37°C and 5% CO_2_ for 24 hours prior to PFAS exposures. PFAS were added to the media at the indicated concentrations and cells were exposed for either 24 hours or 10 days at 37°C and 5% CO_2_. For 10-day exposures, spent media were replaced every 3 days with new PFAS containing media. At the end of the exposures, media were collected and diluted 1:10 in storage buffer (200 mM Tris-HCl pH 7.3 containing 10% glycerol and 1% bovine serum albumin) then frozen at −80°C to test the cytotoxicity. Spheroids were then washed once with Dulbecco’s phosphate-buffered saline (DPBS) (Thermo Fisher Scientific, Franklin, Massachusetts) and lysed with 5–7 µl of TempO-Seq lysis buffer (BioSpyder Technologies Inc, Carlsbad, California). Samples were triturated, incubated for 10 min at room temperature and then stored at −80°C.

### Chemical preparation and exposure conditions

Generation of raw transcriptomic data for single PFAS exposures that were used for comparison of mixture exposure data were the subject of our previous investigations (Reardon *et al.*, 2021; Rowan-Carroll *et al.*, 2021), with the details of PFAS purchase, preparation, and concentration selection for individual PFAS exposures discussed therein. Details of individual PFAS exposures are briefly described herein for clarity. For individual PFAS exposures, the highest concentration of 100 µM was based on the EPA’s ToxCast program’s highest concentration. Additional exposures were selected to capture the response over 3 orders of magnitude and were based on the results obtained for PFOS in our first experiment (Rowan-Carroll *et al.*, 2021). Single PFAS exposure levels were: 0.2, 2, 10, 20, 50, and 100 µM unless otherwise stated (Table 1, bottom).

**Table 1.** List of mixtures, their constituents and concentrations^a^

Mixture name (no. PFAS in mix)	Assigned subgroup of mixture components	Individual PFAS in the mixture	Concentrations (µM)
1 (2)	PFCAs (1)	PFOA +	Total—0.4, 2, 4, 20, 40, 100 (Each—0.2, 1, 2, 10, 20, 50)
	PFSAs (1)	PFOS	
	Other (0)		
2 (9)	PFCAs (6)	PFBA + PFPeA + PFHxA + PFHpA + PFOA + PFNA +	Total—0.18, 1.8, 9, 18, 45, 100 (Each—0.02, 0.2, 1, 2, 5, 11.1)
	PFSAs (3)	PFBS + PFHxS + PFOS	
	Other (0)		
3 (11)	PFCAs (6)	PFBA + PFPeA + PFHxA + PFHpA + PFOA + PFNA +	Total—0.22, 2.2, 11, 22, 55, 100 (Each—0.02, 0.2, 1, 2, 5, 9)
	PFSAs (3)	PFBS + PFHxS + PFOS +	
	Other (2)	6:2 FtS + 8:2 FtS	
4 (11)	PFCAs (8)	PFBA + PFPeA + PFHxA + PFHpA + PFOA + PFNA + PFDA + PFUnA +	Total—0.22, 2.2, 11, 22, 55, 100 (Each—0.02, 0.2, 1, 2, 5, 9)
	PFSAs (3)	PFBS + PFHxS + PFOS	
	Other (0)		
5 (12)	PFCAs (8)	PFBA + PFPeA + PFHxA + PFHpA + PFOA + PFNA + PFDA + PFUnA +	Total—0.24, 2.4, 12, 24, 60, 100 (Each—0.02, 0.2, 1, 2, 5, 8.3)
	PFSAs (3)	PFBS + PFHxS + PFOS +	
	Other (1)	PFOSA	
6 (3)	PFCAs (2)	PFOA + PFNA +	Total—0.9, 9, 18, 30, 60, 100 (Each—0.2, 1, 2, 10, 20, 33.3)
	PFSAs (1)	PFOS	
	Other (0)		
7 (2)	PFCAs (0)		Total—0.4, 2, 4, 20, 40 (Each—0.2, 1, 2, 10, 20)
	PFSAs (0)		
	Other (2)	6:2 FtS + 8:2 FtS	
**Nonmixtures**		**Individual PFAS**	**Concentrations (µM)**
Single PFAS		PFBA, PFPeA, PFHxA, PFHpA, PFNA, PFDA, PFUnA, PFHxS, PFOSA, 6:2 FtS, 8:2 FtS	0.2, 2, 10, 20, 50, 100
		† PFOA, PFOS, PFBS	† 0.02, 0.1, 0.2, 1, 2, 10, 20, 50, 100
		‡ PFUnA	‡ 0.13, 1.3, 6.5, 13, 34, 66

a Mixture names and the total number of PFAS in the mixture are listed in left column. Centre 2 columns list names of PFAS in each mixture arranged by PFAS type. Right column lists exposure concentrations by (top) the total additive molarity of all PFAS in the mixture combined—total, and (bottom) molarity of each individual type of PFAS in the mixture—each. All PFAS mixtures contain equal molarities of each PFAS. Single PFAS (nonmixtures) exposure concentrations are listed at bottom. PFAS indicated with † and ‡ symbols.

**Figure 1.**
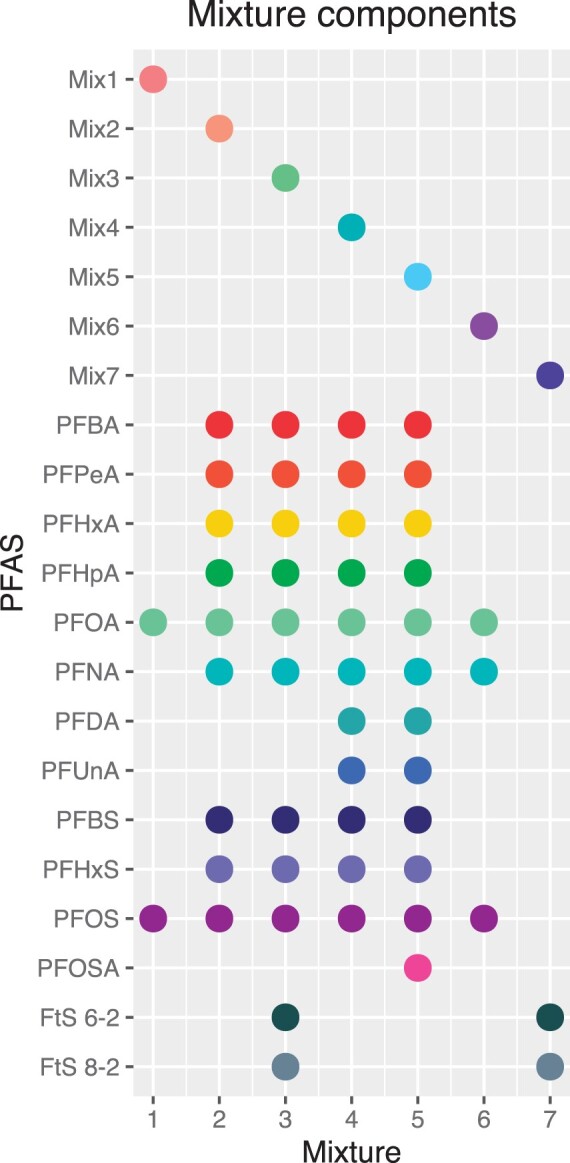
Graphical representation of the components of the 7 PFAS mixtures. Each column represents one mixture denoted by a point at top portion of figure. The components (ie, specific PFAS) used in each mixture are denoted by colored points in the bottom portion of figure. Colors for PFAS and mixtures are maintained across figures throughout article.

Specific mixture concentrations were selected with considerations of reducing operational complexity of exposure experiments while using exposures at a comparable range to single PFAS exposures for computational modeling of concentration-response. All mixtures contained equal molarities of each PFAS in the mixture. Mixtures, exposure concentrations (the total molarity of all combined PFAS in the mixtures) were of a similar range as the single PFAS exposures in our previous studies (Reardon *et al.*, 2021). For each mixture, with the exception of mixture 7, there were 6 exposure concentrations ranging from less than 1 µM up to 100 µM. Mixture 7 had 5 exposure concentrations with a top exposure of 40 µM. Concentration levels were 0.02, 0.2, 1, 2, and 5 µM of each PFAS for high complexity mixtures (mixtures with more than 3 PFAS, mixtures 2–5) and 0.2, 1, 2, 10, and 20 µM for each PFAS for low complexity mixtures (mixtures with 2 or 3 PFAS, mixtures 1, 6, and 7). All mixtures, with the exception of mixture 7, also had a highest concentration level with the total combined molarities of component PFAS summing to 100 µM (Table 1). To allow comparisons of PFAS mixture potencies to single PFAS potencies, all mixture concentrations are reported as total molarity of all PFAS within the mixture. Thus, total combined molarity of PFAS for each mixture exposure level was dependent on the number of PFAS in each mixture; ie, mixture 6, with 3 component PFAS (PFOA, PFOS, and PFNA), had exposures of 0.6, 3, 6, 30, 60, and 100 µM total PFAS (0.2, 1, 2, 10, 20, and 33 µM of each PFAS). See also Table 1 for detailed summary of mixtures and the individual and total molarities of PFAS within the mixtures. See Figure 1 for a graphical depiction of the PFAS included in each mixture.

PFOS (95% purity CAS 1763-23-1), PFOA (95% purity CAS 335-67-1), and perfluorobutanesulfonic acid (PFBS) (95% purity CAS 375-73-5) were purchased from Sigma-Aldrich (Oakville, Ontario). The remainder of PFAS were obtained through a collaboration with the U.S. Environmental Protection Agency (EPA). These PFAS were procured under EPA contract (EP-D-12-034) by Evotec Inc (Bradford, Connecticut) with a minimum target purity concentration of 95%. These were solubilized in dimethyl sulfoxide (DMSO) and monitored to ensure no precipitation was evident. If solubility was not an issue, solutions were prepared at 30 mM; otherwise, stocks were prepared at 10 or 20 mM. EPA PFAS stocks used passed an analytical quality evaluation and were deemed to be stable and free from contaminants (Smeltz *et al.*, 2023) The full list of PFAS used in this study and the abbreviations used throughout the manuscript are summarized in Supplementary Table 1. PFAS were dissolved in DMSO (Sigma-Aldrich, Oakville, Ontario) to prepare working stock solutions up to 30 µM. Final DMSO concentrations in cell culture media were 0.1% for PFAS concentrations below 50 µM, 0.17% for 50 µM and 0.3% for 100 µM PFAS concentrations. PFAS exposures were matched to vehicle only (DMSO) time-matched and concentration-matched controls.

The overall design included twenty 96-well plates (10 plates per timepoint, (24-hour and 10-day exposures)) of liver cell spheroids. For each time point, 4 separate plates contained the complete range of PFAS exposure concentrations (ie, each exposure had one replicate on each of 4 separate plates in the same experiment, resulting in a total of 4 replicates) alongside at least 2 matched DMSO controls (described below). Mixture exposures occurred in the same experiment along with all other PFAS except for PFOS, PFOA, PFBS, and PFDS from Rowan-Carroll *et al.* (2021), which were run separately and had duplicate exposures on each of 2 plates (ie, each exposure had 2 replicates on 2 separate plates in the same experiment, with a total of 4 replicates). Within each plate, there were 10 DMSO controls: 2 at 0.3% for 100 µM PFAS concentrations, 2 at 0.17% for 50 µM PFAS concentrations, and 8 at 0.1% DMSO for all other PFAS concentrations at each timepoint (totaling 8, 8, and 24, respectively, across all plates). DMSO controls for plates with PFOS, PFOA, PFBS, and PFDS exposures totaled 16 at 0.1%, 8 at 0.17%, and 8 at 0.3% for each timepoint. All mention of replicates within the current manuscript and Supplementary Information refer to replicates of PFAS-exposed microtissues (as described above) with independent downstream processing (described below).

### Cytotoxicity assessment and cytotoxicity exclusion criteria

Cytotoxicity was determined using a lactate dehydrogenase (LDH) assay (LDH-Glo, Promega J2380, Madison, Wisconsin), as per the manufacturer’s instructions. Briefly, at the end of PFAS exposures cell culture media were collected and diluted 1:10 in storage buffer (200 mM Tris-HCl pH 7.3 containing 10% glycerol and 1% bovine serum albumin) and frozen at −80°C. For analysis, samples were diluted 1:1 with LDH Detection Reagent and equilibrated for 1 hour at RT before reading relative luminescence units (RLU) (GloMax 96 Microplate Luminometer, Promega Corp, Madison, Wisconsin). Prior to analysis, RLU values from blank controls were subtracted from spheroid media RLU values. LDH ratios for each exposure were calculated by dividing experimental RLUs by the averaged RLU of respective DMSO controls. Means and standard deviations for each set of exposures (chemical, concentration, and timepoint) were then calculated from LDH ratios and expressed as fold change. Sample exclusion due to cytotoxicity was set at a 10-fold increase in LDH over controls (Rowan-Carroll *et al.*, 2021). PFAS exposures at concentrations above the first cytotoxic concentration often resulted in decreased LDH signal, presumably due to cell death and spheroid degradation; therefore, all concentrations higher than the lowest concentrations yielding a 10-fold increase in LDH were also deemed cytotoxic. Samples that were determined to be cytotoxic based on the above metrics were excluded from all downstream analysis in order to ensure the fidelity of subsequent computational quality control of sequencing data (described below).

### TempO-Seq library building and next-generation sequencing

Gene expression was measured using the human TempO-Seq S1500+ panel (House *et al.*, 2017; Mav *et al.*, 2018) (BioSpyder Technologies Inc). This panel of approximately 3000 genes was selected by the National Institute of Environmental Health Sciences to cover a diverse set of biological pathways and provide a representative list of genes that captures transcriptomic variation and toxicological response with a high level of representation when compared to RNA-seq (Bushel *et al.*, 2018; National Toxicology Program, 2015, 2018). For TempO-Seq analysis, liver microtissues were lysed as described above using a volume of 2 × TempO-Seq lysis buffer equal to the residual volume of DPBS. Lysates and positive controls for sequencing reactions (1 × Human Universal Reference RNA—uhrRNA [Agilent Cat. No. 740000] and 1 × Human Brain Total RNA brRNA [ThermoFisher AM7962], as well as 1 × negative controls for sequencing reactions (1 × TempO-Seq lysis buffer alone) were hybridized with detector oligo mix according to the manufacturer’s protocol (Tempo-Seq Human Tox +Surrogate with a Standard Attenuation Transcriptome Kit [96 Samples]) (BioSpyder Technologies, Inc). Positive and negative controls were included on each plate. Hybridization was followed by nuclease digestion of excess oligos, detector oligo ligation, and amplification with tagged primers according to manufacturer’s instructions. During amplification, each sample was ligated to sample-specific barcodes to allow identification of sample sequences after pooled sequencing reactions. Labeled and pooled amplicons were column purified using NucleoSpin Gel and PCR Clean-up kits (Takara Bio USA, Inc, Mountain View, California). Libraries were sequenced in-house, at Health Canada, using a NextSeq 500 High-Throughput Sequencing System (Illumina, San Diego, California) using 50 cycles from a 75-cycle high throughput flow cell.

### Data processing: generation of gene expression data

Data processing was done with R v.3.6.1 (R Core Team, 2022).

To process TempO-Seq data, FASTQ files were generated from the BCL files using bcl2fastq v. 2.20.0.422 (Illumina, San Diego, California). FASTQ files were processed using the TempO-SeqR script v 3.0 provided by BioSpyder (BioSpyder Technologies, Inc), as implemented within our transcriptomics data processing pipeline (https://github.com/R-ODAF/R-ODAF_Health_Canada). Briefly, reads from the FASTQ files were aligned to the TempO-Seq Human Surrogate+Tox Panel (S1500+) v2.0 probes reference sequences (Mav *et al.*, 2018) using STAR 2.7.8a. The qCount function from QuasR (Gaidatzis *et al.*, 2015) was used to extract feature counts specified in a GTF file (provided by BioSpyder) from the aligned reads. The result of this workflow is a table of counts per probe per sample. The gene expression data set is available through the NCBI Gene Expression Omnibus (series numbers GSE145239 and GSE144775).

Sequencing data for individual samples underwent several rounds of quality control for inclusion or exclusion from use in downstream toxicological modeling. The study-wide quality control workflow used in this study was adapted from the recommendations made by Harrill *et al.* (2021) and was used to assess the quality of the alignments and exclude samples if necessary. This data analysis pipeline was an improved implementation of that used in our prior articles (Reardon *et al.*, 2021; Rowan-Carroll *et al.*, 2021) with higher quality control (QC) stringency. Specifically, samples with read counts below 10% of our target depth of 1 M aligned reads (ie, 100 000 reads) were removed; this quality control step flagged both PFAS exposed and control samples. Hierarchical clustering plots were generated (hclust function: default linkage function of hclust function in R; complete-linkage) for all the samples per time point using a distance metric defined as 1-Spearman correlation in order to identify potential outliers. Samples that clustered as singletons when cutting the dendrograms at the 0.1 dissimilarity were removed from the study. We also excluded samples based on the fraction of mapped reads (samples were excluded if the alignment rate was <40%). Additionally, for each sample, we calculated the number of probes with at least 5 uniquely mapped reads; the number of probes required to capture 80% of the signal in a given sample; and the Gini coefficient. For those 3 metrics, any samples identified as outliers based on Tukey’s outer fence (3× interquartile range) were excluded (Harrill *et al.*, 2021). Some samples were excluded based on multiple criteria. Further details on pipeline analysis, QA/QC, and exclusion criteria for all excluded samples are detailed in the Supplementary Rmd File 1 (available at doi:10.5061/dryad.kwh70rz81). Sample exclusion due to cytotoxicity and QA/QC is summarized in Figure 3. A minimum of 2 samples per chemical/mixture for each exposure concentration were required for benchmark concentration modeling (BMC), therefore any exposures with only one sample after QC exclusion were also excluded from BMC analysis.

For downstream analysis (eg, BMD modeling), the count matrix (genes × samples) of all samples passing filters were log_2_-transformed and normalized by their library size scaling factor derived from the median-of-ratios method in DESeq2 to account for differences in the number of reads per sample (Love *et al.*, 2014). To account for differences in the percentage of DMSO used for the dissolution of different chemical concentrations, the log_2_-transformed and DESeq2 normalized data for exposures to higher concentration PFAS that were dissolved in 0.17% and 0.30% DMSO were further normalized to their corresponding DMSO-matched controls and rescaled to the average of the DMSO 0.1% dose group. After normalizing to the controls, the DMSO 0.17% and the DMSO 0.30% controls were then removed.

### Gene-level analysis: benchmark concentration derivation

To generate bootstrap distributions for gene-level and pathway-level analyses, normalized expression data for each gene was bootstrapped 100 times assuming a normal distribution for each concentration group where the mean and standard deviation were based on sample estimates. These data were then used in BMDExpress v3 for BMC modeling (described below) (Buick *et al.*, 2021).

BMC modeling was conducted on log_2_-transformed DESeq2 normalized gene counts using the BMDExpress v3 software package (Phillips *et al.*, 2019), that is freely available at https://github.com/auerbachs/BMDExpress-3/releases. The BMDExpress platform rapidly analyzes large transcriptomic datasets to determine best fit dose response models and calculate benchmark doses at which chemical exposure results in a specified deviation to gene expression. Additional information on BMDExpress and details of BMC analysis is available at https://github.com/auerbachs/BMDExpress-2/wiki (Phillips *et al.*, 2019; Yang *et al.*, 2007).

Bootstrap gene expression data (100 gene expression sets per gene per exposure condition) underwent prefiltering using the Williams Trend Test with 500 permutations, a linear 1.5-fold-change, and *p* ≤ .01 significance to conservatively select probes (ie, genes) with concentration-response behavior. Up or down regulated genes passing Williams Trend Test parameters were modeled using EPA BMDS (United States Environmental Protection Agency, 2022) selected best-fit models (Power, Exp3, Exp5, Linear, and Poly2) with a benchmark response factor of 1 SD, constant variance, and the profile likelihood BMCU/BMCL (upper and lower BMC confidence intervals, respectively) estimation method, to determine concentration-response curves. Best-fit BMCs were automatically selected using BMC, BMCL, and BMCU for each gene with the given parameters (Auerbach* et al.*, 2018). For linear and polynomial models, the Nested Chi-square test was used to determine best-fit models. For this study the gene BMC is the concentration at which the best gene expression response model deviates from the control by 1 SD. BMC data were further filtered to exclude probes with best BMCs that were higher than the highest experimental concentration, with best BMCU/BMCL ratio ≥ 40, or best fit *p-*value of ≤ .1. This process resulted in up to 100 BMCs for each gene for both 24 hour and day 10 exposures. The BMCs derived from BMDExpress were then used to simulate 10 000 experiments for estimation of BMC, BMCL, and BMCU (described below).

Two tPODs were used for potency comparison: (1) the 25th gene BMC (ie, the gene with the 25th lowest BMC); and (2) the lowest Reactome pathway BMC. Gene BMCs passing all filters (from above) were used to simulate independent experiments from which median 25th BMCs and median lowest Reactome pathway BMCs and their respective confidence intervals were derived. To this end, experiments were generated 10 000 times using the R statistical environment by probabilistically selecting genes-BMCs based on their relative frequency. For each experiment, the 25th gene BMC and the lowest REACTOME pathway (Jassal *et al.*, 2020) BMC were recorded resulting in a bootstrap distribution of 10 000 BMCs. For the pathway analysis, the lowest pathway BMC selections required a minimum of 3 genes that represent at least 5% of the pathway. The lowest pathway BMC was the lowest median gene BMC of the altered pathways identified in each experiment. The median and percentile confidence intervals for both of these tPODs were then obtained from the bootstrap distributions.

### Calculation of predicted mixture BMCs and relative potencies

BMC data from BMDExpress (ie, 25th gene BMC and the lowest pathway median BMC) were used for predictions of mixture potency using the concentration addition model (Altenburger *et al.*, 2000; Backhaus *et al.*, 2000; Berenbaum, 1985). In order to derive predicted 25th gene BMCs and predicted BMC accumulation points for mixtures the empirical BMCs for that accumulation point were used for concentration addition calculations. Essentially, concentration addition model or approach applies the difference in relative potency (ie, the concentration of a substance that results in a specific magnitude of change relative to the concentration of another substance that results in the specific magnitude of change) of mixture components and the fractional contribution of each mixture component to calculate a mixture potency. Relative potency factors (RPF_*i*_) were calculated for all PFAS using PFOA as the arbitrary standard according to equation 1, where ECx_std_ is the BMC of the PFAS that is used as the standard (PFOA) that results in the specified effect, and EC*x_i_* is the BMC of each mixture component PFAS *i* resulting in the specified effect.


(1) $${\mathrm{RPF}}_{i}=\frac{{\mathrm{EC}x}_{\mathrm{std}}}{{\mathrm{EC}x}_{i}}$$

To calculate predicted mixture BMCs (ECx_mix_) that result in the specified effect, the standard concentration addition equation (equation 2) (Altenburger *et al.*, 2000; Backhaus *et al.*, 2000) was simplified for equimolar mixtures with (priorly calculated) RPF_*i*_ to equation 3. *n_i_* is the number of equimolar component PFAS (*i*) in the mixture. *p_i_* is the fraction (molarity of component *i*/sum of molarities of all components) of component *i* in the mixture. For all comparisons fold differences are the ratio of the compared values.


(2) $${\mathrm{EC}x}_{\mathrm{mix}}={\left(\sum_{i=1}^{n}\frac{p_{i}}{{\mathrm{ECx}}_{i}}\right)}^{-1}$$

(3) $${\mathrm{EC}x}_{\mathrm{mix}}=\frac{{\mathrm{EC}x}_{\mathrm{std}}}{\sum_{i=1}^{n}{\mathrm{RPF}}_{i}}\times n_{i}$$

## Results

###  

#### Cytotoxicity assessment

**Figure 2.**
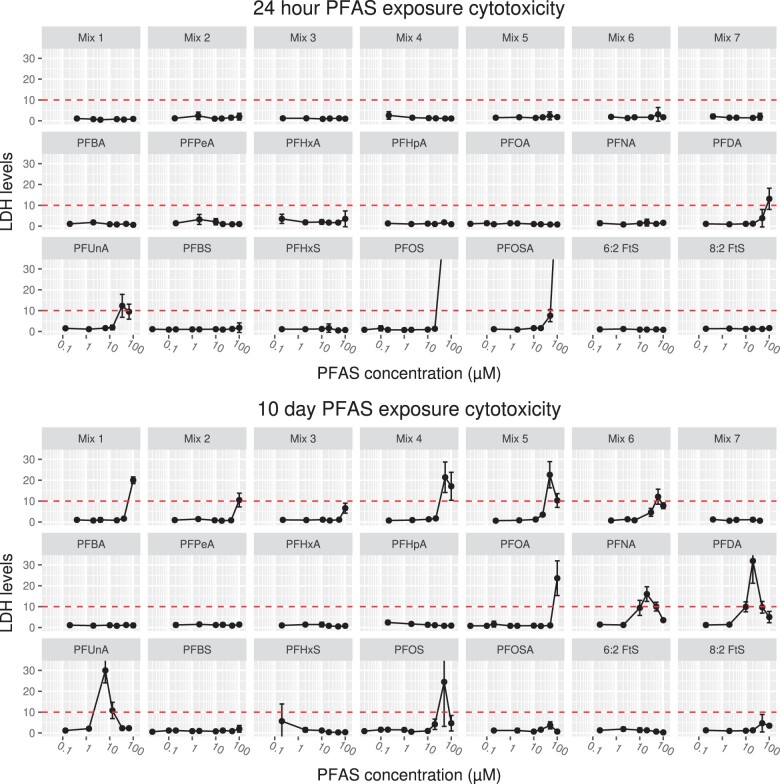
Cytotoxicity determination of PFAS exposures using lactose dehydrogenase (LDH). Relative mean LDH levels (luminescence) between DMSO control and PFAS exposed liver spheroid media following 24-hour (top) and 10-day (bottom) exposures expressed as fold change over control. Red dashed lines indicate the 10-fold cytotoxicity threshold applied in our study. Exposures are indicated by black dots. Exposure concentrations vary between mixture and single PFAS exposures (see Table 1). Mixture exposure levels are indicated as total additive molarity of all PFAS in mixtures. Mixture and single PFAS exposures are indicated at top of figure facets. Error bars indicate standard deviation of 4 exposures (*n* = 4).

**Figure 3.**
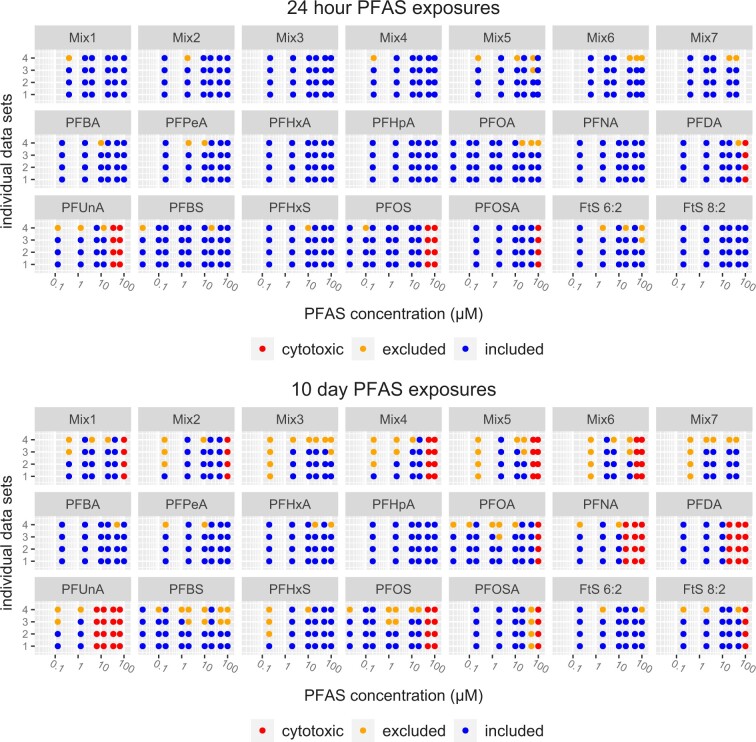
Graphical depiction of experimental setup and inclusion or exclusion of individual PFAS exposure data in downstream analysis. Graphical depictions of inclusion and exclusion of individual PFAS exposure data in downstream analysis for 24-hour (top) and 10-day exposures (bottom). Individual points represent single PFAS exposures and their exposure concentrations. PFAS exposures that were removed due to cytotoxicity are depicted in red. PFAS exposures that were excluded due to R-ODAF quality control failures are depicted in orange. PFAS exposures that passed all R-ODAF quality control parameters and were used for downstream analysis are depicted in blue. Facet titles at top of each graph indicate the PFAS or mixture studied. Points are aligned to their corresponding exposure concentrations with mixtures and PFAS having varying exposures. All experimental exposures are indicated.

At 24 hours, no PFAS mixtures caused cytotoxicity; however, several medium to long-chain individual PFAS (PFDA, PFUnA, PFOS, and PFOSA) were cytotoxic at the highest concentration (100 µM) (Figure 2). PFUnA was also cytotoxic at 20 µM at 24 hours. In contrast to 24 hour results, 10-day exposures caused 10-fold increases in LDH for several single PFAS and PFAS mixtures (Figure 2). Mixtures 1 and 2 were cytotoxic at 100 µM, while mixtures 4, 5, and 6 were cytotoxic at their 2 highest concentrations. Cytotoxicity data for single PFAS were previously reported (Reardon *et al.*, 2021; Rowan-Carroll *et al.*, 2021), but are included here for clarity as transcriptomic data for single PFAS exposures have been reanalyzed for this study starting from raw sequence data and used for the present analysis of mixture potency. Cytotoxicity results were solely used as a quality control measure for removal of samples that would have a confounding effect on computational transcriptomic quality control. Samples excluded due to cytotoxicity are depicted in Figure 3. Analysis of cytotoxicity data is outside of the scope of this article.

#### TempO-seq data processing and quality control

Sequencing reads for both mixtures and single PFAS were analyzed using an updated pipeline derived from our previous work (Reardon *et al.*, 2021; Rowan-Carroll *et al.*, 2021) that implements elements of the Regulatory Omics Data Analysis (R-ODAF) framework (Verheijen *et al.*, 2022). Our analysis of sequencing quality control metrics confirmed that signals were negligible in no-spheroid controls and that the reference RNA samples showed high intra- and interplate reproducibility (interplate average spearmen coefficient of 0.973), confirming TempO-Seq technical quality. Median read depth for samples was 1 392 334 mapped reads. While the minimum QC cutoff was 100 000 reads, the minimum number of mapped reads for samples that passed all other QC metrics was 389 717. The maximum number of reads for any passing spheroid sample was 3 712 291. The fraction of mapped reads for retained samples ranged from 0.40 to 0.78. Samples excluded due to data processing quality control are depicted in Figure 3. Additional details of data processing and quality control are available in the Supplementary Rmd File 1 (available at doi:10.5061/dryad.kwh70rz81).

#### Benchmark concentration determination

**Figure 4.**
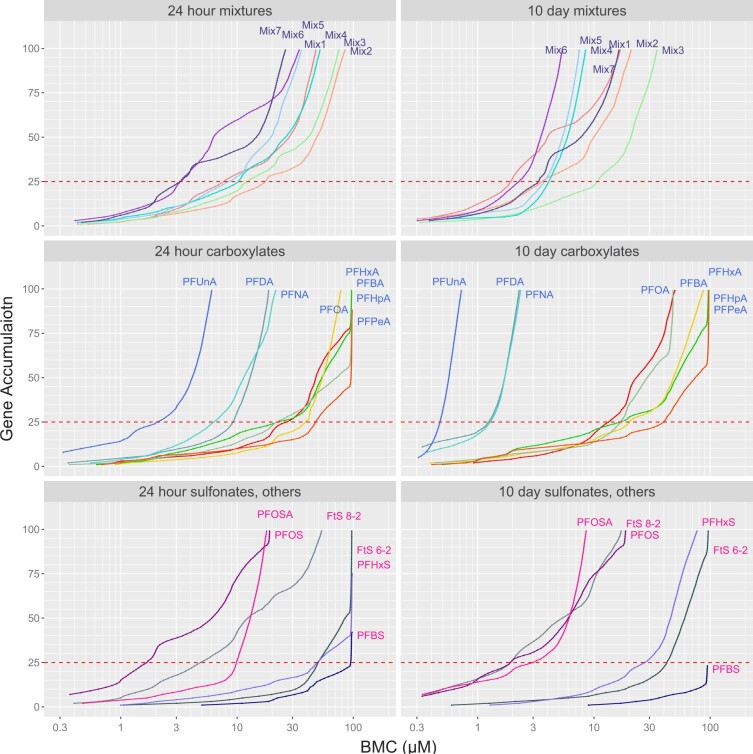
Accumulation plots of gene BMC responses. Accumulation graph of the lowest 100 gene BMCs of PFAS-exposed liver spheroids shown for PFAS mixtures and single PFAS. Note that the top of the line ends at the total number of genes fitting BMCs if there were <100 BMCs. 24-hour (left column) and 10-day exposures (right column) are divided into mixtures (top row), carboxylate-type PFAS (center row), and sulfonate and other types of PFAS (bottom row) for presentation. Accumulation graphs show number of genes with a concentration response of one standard deviation over control (BMCs) at all PFAS concentration levels as medians of 10 000 bootstrap experiments for PFAS mixtures and single PFAS. The red dashed line indicates the 25th gene BMC tPOD for each chemical or mixture.

To visualize gene expression changes in response to increased PFAS concentrations, gene accumulation plots of the BMCs for each PFAS/mixture and timepoint were plotted and ranked from the lowest to highest gene BMC (ie, the gene with the lowest BMC is ranked 1, the next higher BMC is 2, etc.,) up to the 100th gene BMC (Figure 4). For most PFAS and mixtures the accumulation plots were largely flat or had very small slopes across the concentration range up to an “initiation” point on the accumulation curve (Figure 4). This point has been referred to as the “concerted molecular response” (Johnson *et al.*, 2022) and reflects the concentration at which transcriptional changes begin to broadly increase and impact the cells. Once initiated, BMCs for each PFAS and mixture were closely spaced by concentration and accumulation generally continued in a linear or exponential fashion (Figure 4 and Supplementary Figure 1). Some lower potency PFAS did not have 100 genes fitting BMC models. These PFAS (PFBS, PFHxS, PFPeA, and PFHpA at 24 hours and PFBS at 10 days) weakly affected gene expression and their highest accumulated BMCs approached their highest exposure concentrations of 100 µM (Figure 4).

#### 25th gene BMC transcriptomic point of departure

Previously, we proposed that the 25th gene BMC is a strong predictor of the concentration at which the “concerted molecular response” in a transcriptomic data set occurs and provides a conservative tPOD (Matteo *et al.*, 2023; Reardon *et al.*, 2021). Thus, an analysis was done specifically assessing the effects of PFAS and PFAS mixtures on the 25th gene BMC.

**Figure 5.**
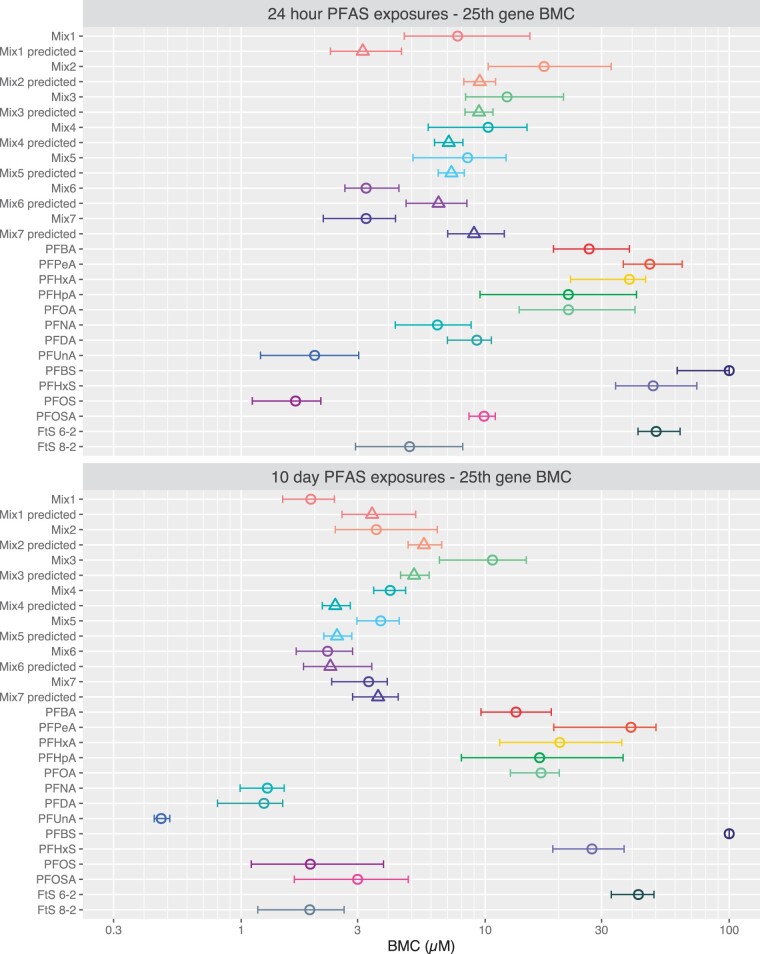
Empirical and predicted BMCs for each mixture based on concentration addition for the 25th gene BMC tPOD. The 25th lowest ranked gene BMC tPOD for each single PFAS exposure, mixture, and predicted concentration based on concentration addition of mixture components is shown. Empirical BMC values are indicated by open circles. Predicted BMC values are indicated by open triangles. Values of single PFAS and mixtures are median values of 10 000 bootstrap experiments. 95% confidence intervals that reflect the center range of 95% of 10 000 BMDExpress bootstrap experiments are shown.

The 25th gene BMCs and their confidence intervals were used to compare potencies (Figure 5) and produce RPFs for each PFAS (Supplementary Table 2). PFBS exposures resulted in few BMCs; therefore, the 100 µM exposure limit of our experimental design was used as the BMC for PFBS for both 24-hour and 10-day exposures. All other PFAS and mixtures had 25th gene BMCs within their exposure concentration ranges.

Potency for every PFAS and mixture, with the exception of mixture 7 and PFOS, increased from 24-hour to 10-day exposures (Figure 5 and Supplementary Table 2). However, the relative potencies of individual PFAS and mixtures were largely maintained across timepoints, with many PFAS increasing in potency to a similar degree. Longer chain carboxylate-type PFAS had higher relative decreases in 25th gene BMCs between time points than shorter chain, with PFNA (C9), PFDA (C10), and PFUnA (C11) BMCs decreasing by 5.0-, 7.5-, and 4.3-fold, respectively. In contrast, shorter-chain carboxylate-type PFAS 25th gene BMCs declined by 2.0-, 1.2-, 1.9-, 1.3-, and 1.3-fold from 24 hours to 10 days for PFBA (C4), PFPeA (C5), PFHxA (C6), PFHpA (C7), and PFOA (C8), respectively (Figure 5 and Supplementary Table 2). These differences resulted in a greater distinction of the 25th gene BMCs between shorter and longer chain PFAS at day 10. Longer exposures to sulfonate-containing PFAS resulted in relatively smaller decreases in their 25th gene BMC from 24 hour to day 10 exposures as compared to most carboxylate PFAS.

To determine if PFAS mixtures have differing potencies compared to their individual components, the concentration addition paradigm was used to predict mixture potency. Specifically, the empirical 25th gene BMCs for mixtures were compared to mixture BMCs predicted based on the concentration addition paradigm calculated from the 25th gene BMCs of their individual constituent PFAS. Predicted mixture 25th gene BMCs were within ± 2-fold of empirical values, with the exception of mixtures 1 and 7 at the 24 hour time point, which were both within ± 3-fold of empirical values (Figure 5, Supplementary Table 1 and Excel File 1). Half of the empirical and predicted BMCs had overlapping 95% confidence intervals indicating no significant differences between them. However, at 24 hours, mixtures 1, 6, and 7 had nonoverlapping confidence intervals; mixture 1 was 2.5-fold less potent than its predicted value, and mixtures 6 and 7 were 2.0- and 2.8-fold more potent than their predicted 25th gene BMCs, respectively. At 10-day exposures, mixture 1 was conversely 1.8-fold more potent than its predicted value while mixtures 3, 4, and 5 were 2.1-, 1.7-, and 1.5-fold less potent than suggested by concentration addition. When the upper and lower bounds of empirical and predicted BMC CIs are used to determine fold differences (ie, the upper bound of the lowest potency BMC CI compared to the lower bound of the highest potency BMC CI), no mixture had a greater than 2-fold difference in potency. Only mixture 4 (1.25-fold) at 10 days and mixture 7 (1.67-fold) at 24 hours had a higher than 1.1-fold difference in potency. Overall, mixture BMCs followed their predicted BMCs. Differences between empirical and predicted PFAS mixture BMCs that had nonoverlapping CIs were low in magnitude and there was no clear trend for differences between empirical and predicted BMCs.

To determine if mixture predictions remained consistent through a range of BMCs, predicted BMCs were also calculated for mixtures at each accumulation point up to the highest relative standard BMC (PFOA) for the 24 hour time point, or the 100th gene BMC for the 10-day exposure. Overall, empirical and predicted BMCs for mixtures were comparable throughout the accumulation curve (Supplementary Figure 2).

#### Lowest pathway median point of departure

The lowest pathway median BMC for each PFAS or mixture, consisting of the lowest median BMC of any pathway with at least 3 genes fitting BMC models and comprising at least 5% of the pathway, was used as a second tPOD to explore whether PFAS adhere to the dose additivity model. This tPOD was selected based on recommendations from an expert panel (National Toxicology Program, 2018). This analysis was done using BMCs derived from the bootstrap gene expression data and statistical cutoffs as above and using the REACTOME pathway database (Jassal *et al.*, 2020). Mixture lowest pathway median BMCs were derived from empirical data of individual PFAS and were predicted through concentration addition calculations using median pathway BMCs for mixture constituent PFAS.

**Figure 6.**
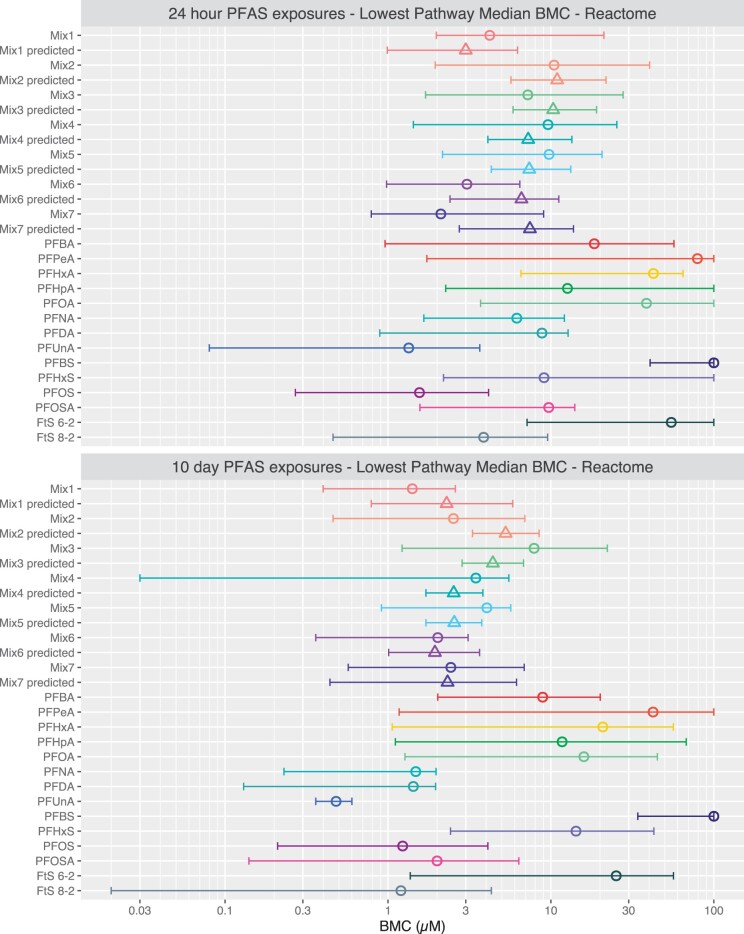
Empirical and predicted BMCs for each mixture based on concentration addition for the lowest pathway median BMC tPOD. The lowest pathway median BMC tPOD for each single PFAS exposure, mixture, and predicted BMC based on concentration addition of individual mixture components are shown. Empirical BMC values are indicated by open circles. Predicted BMC values are indicated by open triangles. Values of single PFAS and mixtures are median values of 10 000 bootstrap experiments. 95% confidence intervals are shown to indicate the center range of 95% of 10 000 BMDExpress bootstrap experiments.

Lowest pathway median BMCs corresponded closely to 25th gene BMCs for single PFAS, PFAS mixtures and for predicted PFAS mixtures (compare Figs 5 and 6 gene and pathway BMCs in Supplementary Table 1). Confidence intervals for lowest pathway median BMCs were large for all mixtures and PFAS, and confidence intervals for empirical and predicted BMCs consistently overlapped for all mixtures.

## Discussion

An important consideration for toxicological assessment is to determine if PFAS in mixtures interact to cause synergistic effects, which may necessitate more conservative exposure limits. The present study applied high-throughput transcriptomics to derive tPODs reflecting the PFAS concentration at which the cells exhibit a “concerted molecular response” (Johnson *et al.*, 2022) in human liver cell spheroids. These tPODs were used to assess the potency of PFAS mixtures and determine the accuracy of the principal of concentration addition in predicting mixture potency. Transcriptomic PODs derived from percentiles (eg, 5th percentile) selected from the transcriptomic BMC distributions are more likely to be influenced by study design parameters such as exposure concentration selections (eg, highest exposure tested or interval between highest noncytotoxic exposure and cytotoxic response), which may affect total BMC accumulation number. Thus, herein the 25th gene and lowest pathway BMCs were chosen as more robust tPODs to represent the “concerted molecular response” (Matteo *et al.*, 2023; Reardon *et al.*, 2021). Analysis of the 25th gene BMC tPOD revealed that the potencies of PFAS mixtures are largely predictable using the standard concentration addition model (Berenbaum, 1985). This conclusion is based on the findings that the majority of PFAS mixture empirical and predicted BMCs had overlapping 95% confidence intervals. Our work is also consistent with other studies proposing that a 2-fold difference threshold from the concentration additional model provides an acceptable, slightly conservative model for evaluating mixtures (Belden *et al.*, 2007; Martin *et al.*, 2021).

Four of the mixtures within this study (mixtures 2–5) were of high complexity, containing 9–12 PFAS, including long- and short-chain PFAS as well as sulfonate and carboxylate type PFAS. Mixture 4 contained the same PFAS as mixture 2, but with 2 additional long-chain carboxylate PFAS: PFDA and PFUnA. Mixture 3 was identical to mixture 2 with 2 additional chemicals: 6:2 FtS and 8:2 FtS. Mixture 5 was identical to 4, but with the addition of PFOSA. Predicted 25th gene BMCs for mixtures 2 and 3, and for mixtures 4 and 5, were highly similar to each other for each timepoint. As expected, the addition of the more potent PFAS, PFDA, and PFUnA, within mixtures 4 and 5, resulted in higher predicted potencies relative to mixtures 2 and 3. At 24-hour exposures, all high complexity mixtures had overlapping 95% confidence intervals for empirical and predicted 25th gene BMCs, with ratios less than 2-fold. For 10-day exposures, predicted BMCs were within ±2-fold of empirical BMCs, with the exception of mixture 3 that had an empirical 25th gene BMC that was 2.1-fold higher than that predicted by concentration addition. mixtures 3, 4, and 5 had predicted 25th gene BMCs that were lower than their corresponding empirical BMCs (nonoverlapping confidence intervals), potentially offering some evidence of weak antagonism. The reasons for this apparent trend toward antagonism within these mixtures is unclear; however, the 3 most potent PFAS following 10-day exposures, PFNA, PFDA, and PFUnA, all had 3 or fewer concentrations that passed cytotoxicity and transcriptomic QC measures that could be used for estimating BMCs. With fewer concentrations for modeling, this may decrease the accuracy of the 25th gene BMCs that were used for concentration addition calculations for mixtures containing these PFAS (Ewald *et al.*, 2022). Overall, our analysis revealed no strong evidence of synergistic interactions of individual PFAS within these complex mixtures and supports that a concentration addition model tPOD would be conservative.

Three mixtures were of low complexity, containing 2 or 3 PFAS. Mixture 1 contained PFOA and PFOS, and mixture 6 PFOA, PFOS, and PFNA. The predicted 25th gene BMC for mixture 6 was strongly influenced by (the addition of) PFNA, which had an approximately 5-fold increase in potency between 24-hour and 10-day exposures and resulted in a large increase in predicted potency for mixture 6. For 24-hour exposures, mixture 1 trended toward antagonism while mixture 6 trended toward synergy, with 2- and 2.5-fold differences between empirical and predicted 25th gene BMCs, respectively. It is unclear how the addition of PFNA, which is chemically highly similar to PFOA but has a potency similar to PFOS, might cause a shift from antagonism in mixture 1 to synergy in mixture 6. Nonetheless, by 10 days of exposure, the trend for mixture 1 was opposite to its 24 hour trend and inverted toward synergy, with nonoverlapping confidence intervals and a 1.8-fold difference between its 25th gene empirical and predicted BMCs. In contrast, by day 10 mixture 6 had highly similar predicted and empirical BMCs with no evidence of interactions among mixture components. Mixture 7, which had one high potency PFAS, 8:2 FtS, and one low potency PFAS, 6:2 FtS, had the strongest trend for synergy of all PFAS mixtures at 24 hours, with a 2.8-fold difference between predicted and empirical 25th gene BMCs; however, similarly to mixture 6, by 10 days of exposure the predicted and empirical BMCs were highly similar. Thus, as with the high complexity mixtures tested, the concentration addition model provides an accurate estimate of low complexity mixture potency, and evidence of synergy or antagonism is not more apparent in these mixtures as compared to high complexity mixtures.

To broaden the above findings beyond the 25th gene, accumulation plots of both empirical and predicted BMCs for all mixtures and timepoints were derived up to the 100th ranked gene BMC. The empirical and predicted accumulation BMC curves are remarkably similar. Our results suggest that addition model holds strong across a variety of potential BMC-rank tPODs. Therefore, this more expanded analysis suggests that antagonism or synergy will not become more apparent in selecting higher (or lower) BMCs from the distribution.

We also examined the use of the lowest pathway median BMC as a tPOD for analysis of PFAS mixture potency using concentration addition. Overall, 25th gene BMCs and lowest pathway median BMCs aligned well; these tPODs were within ± 2-fold for all PFAS at both timepoints with only one exception. However, the 95% confidence intervals for the lowest pathway median BMCs were far larger than for 25th gene BMCs, with many confidence intervals being over an order of magnitude. This increased uncertainty for pathway tPODs may be due to some of the extremely low BMCs being derived from concentration-response curves that were asymptotic to the control. Thus, we have less confidence in these extremely low BMCs. Additionally, the number of genes within pathways may also influence pathway medians with potential for low median BMCs in pathways with few genes, and medians BMCs for pathways with high numbers of genes being influenced by the total overall number of BMCs. Because this approach is based on the median BMC, there may be pathway median genes with very high BMCs (ie, relatively nonresponsive) that will contribute to large confidence intervals. Our results indicate that the 25th gene BMC tPOD provides higher precision for comparison of chemical potencies relative to the lowest gene set (pathway) BMC approach.

Prior work has shown synergism and antagonism of some PFAS combinations. Studies using endpoints such as lethality in animal models (Ding *et al.*, 2013; Rainieri *et al.*, 2017), and cytotoxicity in cellular models (Hu *et al.*, 2014; Ojo *et al.*, 2020; Rainieri *et al.*, 2017), are informative and important; however, the concentrations used in these studies have generally been very high and overtly toxic and thus may lack the sensitivity needed for informing regulatory decisions. Work focused on highly sensitive PFAS-target nuclear receptor activation has also generated mixed findings and interpretations (Carr *et al.*, 2013; Kjeldsen and Bonefeld-Jørgensen, 2013; Ojo *et al.*, 2022; Wolf *et al.*, 2014). A study of interest, using a PPARα reporter system, had similar conclusions to ours, with low concentration binary PFAS mixtures having potencies predictable by concentration addition (Wolf *et al.*, 2014). A recent in-depth analysis of maternal and neonatal effects in rats at various dose levels also found that mixtures of PFOA and PFOS had effects predictable by dose addition for nearly all measures assessed (Conley *et al.*, 2022). Through use of a nontargeted *ex-vivo* system that aims to closely parallel human biology and is capable of capturing PFAS effects regardless of mode of action, our data also suggest that the PFAS examined in our study act additively to elicit a transcriptional response at low concentrations.

Also of note, the relative potencies between PFOA, and many of the other PFAS used in our mixture study, closely match those based on an analysis of rat liver data (Bil *et al.*, 2021). However, we also found that some short-chain PFAS (PFBA [C4] and PFHxA [C6]) had 10–50 times higher relative potencies in our microtissue exposures than in Bil *et al.*’s analysis of *in vivo* exposure data, underscoring the importance of accurate *in vitro* to *in vivo* extrapolation for cell-based toxicological profiling of PFAS. Such extrapolations are beyond the scope of the present study and will be the subject of future work.

The goal of the present study was 2-fold: (1) to further advance a NAM allowing unbiased, highly sensitive, and human relevant assessment of toxicant exposures; and (2) to assess (and inform prediction of) the effects of PFAS mixture exposures. Our multidonor human liver microtissue tPOD model of PFAS toxicity allows observation of effects that capture sensitive molecular changes that are likely directly related to physiological responses. Based upon comparison of empirical mixture exposure data to predictions derived from single PFAS exposure data, we show that PFAS mixtures largely act as expected according to the principal of concentration addition for the PFAS studied herein. Together with our previous work (Reardon *et al.*, 2021; Rowan-Carroll *et al.*, 2021), this research advances PFAS assessment by providing foundational data to support read across and predictions of the effects of PFAS in mixtures.

## Supplementary Material

kfad044_Supplementary_Data

## Data Availability

The data sets used in this article are publicly available at: https://www.ncbi.nlm.nih.gov/geo/query/acc.cgi?acc=%20GSE145239%20and%20GSE144775.

## Notes

Dryad Digital Repository DOI: http://doi.org/10.5061/dryad.kwh70rz81
